# Buccal Peri-Implant Soft Tissue Augmentation by Means of a Porcine Collagen Matrix: A Proof of Concept Technical Note

**DOI:** 10.3390/ma14010093

**Published:** 2020-12-28

**Authors:** Mario Beretta, Carlo Maiorana, Mattia Manfredini, Susanna Ferrario, Pier Paolo Poli

**Affiliations:** Maxillofacial Surgery and Odontostomatology Unit, Implant Center for Edentulism and Jawbone Atrophies, Fondazione IRCCS Ca’ Granda Ospedale Maggiore Policlinico, University of Milan, Via della Commenda 10, 20122 Milan, Italy; dr.marioberetta@gmail.com (M.B.); carlo.maiorana@unimi.it (C.M.); mattiamanfredinidr@gmail.com (M.M.); ferrariosusanna@gmail.com (S.F.)

**Keywords:** biomaterials, collagen matrix, dental implants, peri-implant plastic surgery, soft tissues, xenograft

## Abstract

The quality and quantity of peri-implant soft tissues at the crestal portion of dental implants are important aspects to consider for a long-term successful implant-supported rehabilitation. Some relevant factors attributed to the implant health include mucosal thickness and keratinization. In this respect, many techniques and materials have been described to augment and improve buccal peri-implant soft tissues. Over the last few years, newly developed xenogeneic collagen matrices have been introduced in peri-implant plastic surgery to replace autogenous soft tissue grafts; however, data remain controversial so far. Thus, the purpose of the present report was to present a novel surgical technique conceived to augment buccal peri-implant soft tissues in combination with a volume-stable porcine collagen matrix. The rationale and the fundamental concepts that led to the use of a xenogeneic matrix to increase soft tissue volumes were also discussed.

## 1. Introduction

Periodontal and peri-implant plastic surgery procedures are well-known surgical techniques mainly indicated to increase the volume of the soft tissues and the amount of the keratinized mucosa in order to ensure long-term tissue stability. Since 1995, the importance of an adequate amount of keratinized peri-implant mucosa to maintain the implant health has been underlined. The authors have associated soft tissue deficiency with increased plaque accumulation, increased gingival recessions and a significant loss of attachment compared to implants with an adequate thickness of keratinized gingiva [1]. Nowadays, in case of volumetric defects at the buccal surface of the peri-implant mucosa, soft tissue augmentation surgery is strongly recommended and considered part of the implant treatment [2]. In this respect, peri-implant soft tissue augmentation by means of autogenous connective tissue graft (CTG) can be performed at different timing during the treatment, with no significant differences in terms of peri-implant mucosal thickness gain [3]. Tissue biotype and aesthetic expectations are key factors in the decision-making process for soft tissue augmentation. Indeed, a thin peri-implant tissue biotype seems to be more susceptible to recessions and may compromise the function and aesthetics of the rehabilitation in the long term [4,5,6]. Additionally, soft tissue grafting contributes to more than 40% of the final tissue volume and may lead to more stable marginal peri-implant bone levels over time [7,8]. In 2015, Linkevicious et al. suggested that vertical gingival thickness is critical in preventing bone resorption. Indeed, thick mucosal tissue proved to be a positive predictive factor for peri-implant bone stability [7]. A potential reason could be related to the resultant better mucosal seal that would limit the propagation of oral pathogens at the implant–abutment interface close to the bone level [9,10].

So far, different grafting materials have been described in literature for soft tissue augmentation around dental implants, ranging from autogenous grafts to allografts, xenografts and synthetic substitutes [11]. Notable among the biomaterials are the collagen matrices (CMs), which have numerous benefits compared to other soft tissue grafts, including less post-operative pain and higher patient satisfaction compared to autogenous graft [12]. However, to the best of the authors’ knowledge, the current evidence regarding recently developed porcine CMs in the treatment of soft tissue deficiency at the implant site is still lacking. Therefore, the aim of the present study was to describe a novel surgical technique in the management of three-dimensional soft tissue defects combined with a new dedicated porcine CM as an alternative to autogenous CTG, in order to improve the stability of the augmented mucosa.

## 2. Case Report

### 2.1. Material

In the present case report, a newly developed CM (Geistlich Fibro-Gide^®^, Geistlich Pharma AG, Wolhusen, Switzerland) has been used as a soft tissue substitute. The said biomaterial consists in a volume-stable porcine, highly porous, and resorbable CM, designed for soft-tissue augmentation. The CM is made of reconstituted collagen that undergoes smart chemically cross-linking to improve its volume stability while maintaining a good biocompatibility. This is made possible by the porous structure of the CM which allows blood clot stabilization and the ingrowth of host cells. More in detail, the CM is made of 60–96% (*w/w*) porcine collagen type I and III and 4–40% (*w/w*) elastin, has an average pore diameter of 92 µm, and a volume porosity of 93% with interconnected pores. The porous network of the CM supports angiogenesis, formation of new connective tissue, and stability of the collagen network in submerged healing situations. The CM can be applied either in a dry or wet state based on individual preference. Pre-wetting can be done with the patient’s own blood or sterile saline solution. It should be noted that the device transiently gains approximately 3–12% in each dimension upon wetting. This must be taken into account when defining the final dimension to allow tension-free wound closure. In this respect, the CM can be adjusted in size and thickness, both in wet and dry state. According to the manufacturer’s instruction, a scalpel is recommended to use when in dry state and scissors when in wet state.

### 2.2. Case Presentation

A 47-year-old non-smoking healthy male patient presented to the authors’ attention seeking for a fixed implant-supported rehabilitation to replace a missing upper first molar. The medical history was noncontributory.

The preliminary clinical and radiological examinations showed the presence of an adequate amount of pristine bone associated with a buccal soft tissue contraction in the horizontal dimension. The second level radiological evaluation by means of a cone-beam computed-tomography scan confirmed the presence of sufficient bone to place the implant in a prosthetically driven position without recurring to additional bone regeneration procedures. A signed informed consent was obtained from the subject prior to commencement of the therapy. All surgical and prosthetic procedures were performed according to the principles outlined in the Declaration of Helsinki on experimentation involving human subjects as revised in 2013.

**Figure 1:**
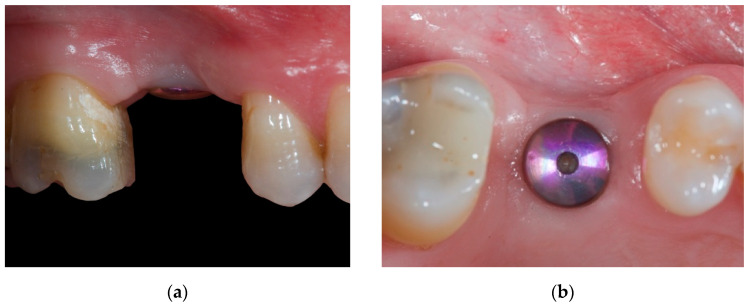
Intraoral view of the soft tissues after a 3-week healing period from the uncovering procedure: (**a**) Lateral view. (**b**) Occlusal view.

Implant positioning was performed with the aid of a surgical guide based on a preliminary analogic prosthetic wax-up of the missing natural element. Implant bed preparation was carried out and a rough-surfaced implant (Oxyimplant Psk Biomec s.r.l. Colico, LC, Italy) was inserted according to the manufacturer’s instructions. After 4 months of submerged healing, the second stage surgery was performed to uncover the implant and connect the healing abutment. A mucoperiosteal flap was designed with the crestal incision displaced slightly palatally in order to reposition an adequate amount of keratinized gingiva at the buccal aspect of the implant. Soft tissues were left to heal for 3 weeks (Figure 1a,b). At this point, a scan body was connected to the implant and a digital impression was taken (CS 3600, Carestream Dental^©^, Carestream Health, Rochester, NY, USA).

**Figure 2:**
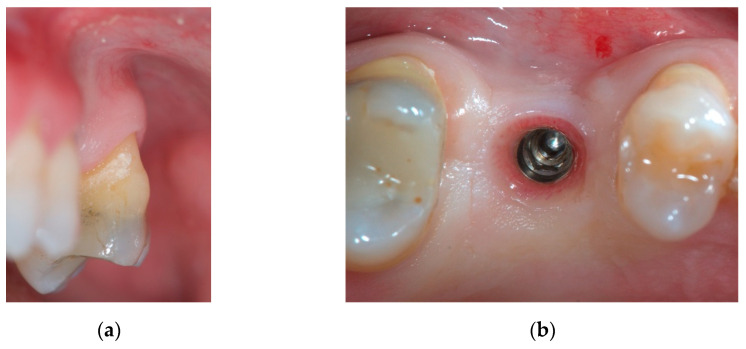
Intraoral view of the soft tissues before the peri-implant plastic surgery procedure: (**a**) Buccal view. (**b**) Occlusal view.

After an additional week (Figure 2a,b), soft tissue augmentation was carried out by means of a volume-stable porcine resorbable CM simultaneously with the delivery of the temporary prosthesis. The emergence profile of the interim crown was customized with an ideal shape in order to support the new volumes. The surgical procedure consisted in a mixed split- and full-thickness flap.

**Figure 3:**
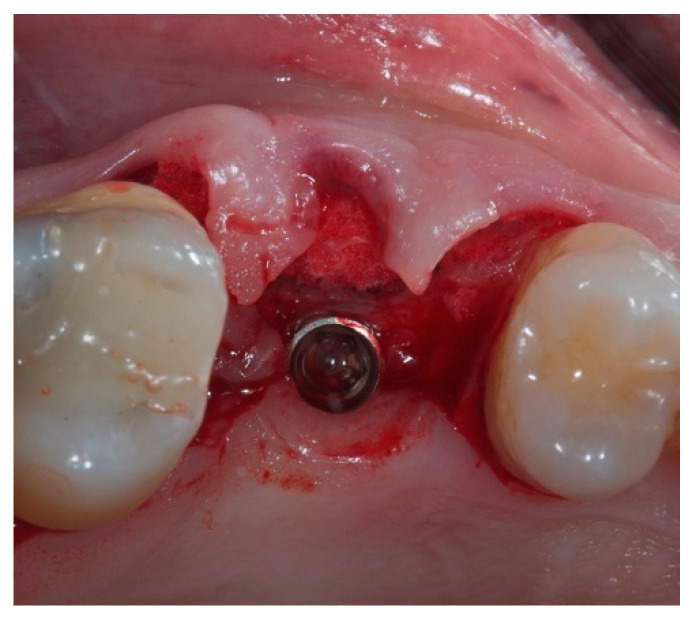
Intraoral occlusal view of the flap design. The collagen matrix is visible at the buccal aspect of the implant inside the soft tissue envelope soaked by the blood clot.

Flap design involved the split-thickness elevation of the mesial and distal papilla, anticipating the buccal displacement of the papillae as a result of the increased final buccal volume. Once the buccal bone margin was reached, the split-thickness incision shifted to a full-thickness design. In this way, a mucoperiosteal envelope was created avoiding releasing incisions up to the muco-gingival junction, in order to obtain a three-dimensional space intended to be filled by the CM. At this point, the CM was shaped in order to fit the missing volume in its dry state with a surgical scalpel blade No.15C, and was carefully placed inside the envelope until the untouched periosteum was reached apically. Thus, the recipient bed of the CM was represented palatally by the preserved buccal bone wall, and buccally by the periosteum covering the inner portion of the envelope. As the apical border of the envelope was delimited by the untouched periosteum at the level of the mucogingival junction, and the coronal border was sealed by the emergence profile of the temporary crown, there was no need to suture the CM (Figure 3).

**Figure 4:**
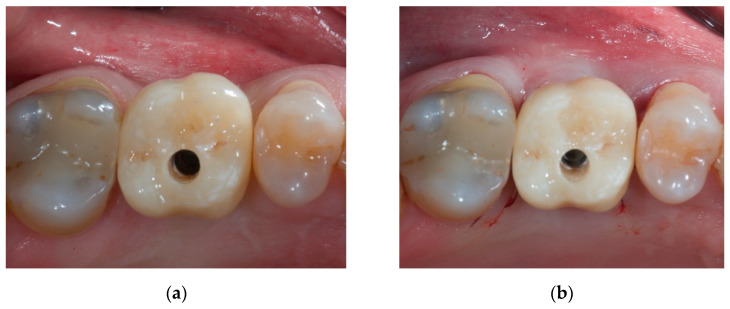
Occlusal view of the surgical site and the temporary screw-retained crown highlighting the volumetric differences between pre-augmentation (**a**) and post-augmentation (**b**) in the immediate post-operative period.

Once the CM was placed inside the envelope, the suture (6-0 polyglycolic acid) had the only role of repositioning the mesial and distal papilla apically according to the augmented buccal volume. The temporary resin crown was finally screwed to the implant in order to sustain the volumetric increase of the soft tissues (Figure 4a,b).

**Figure 5:**
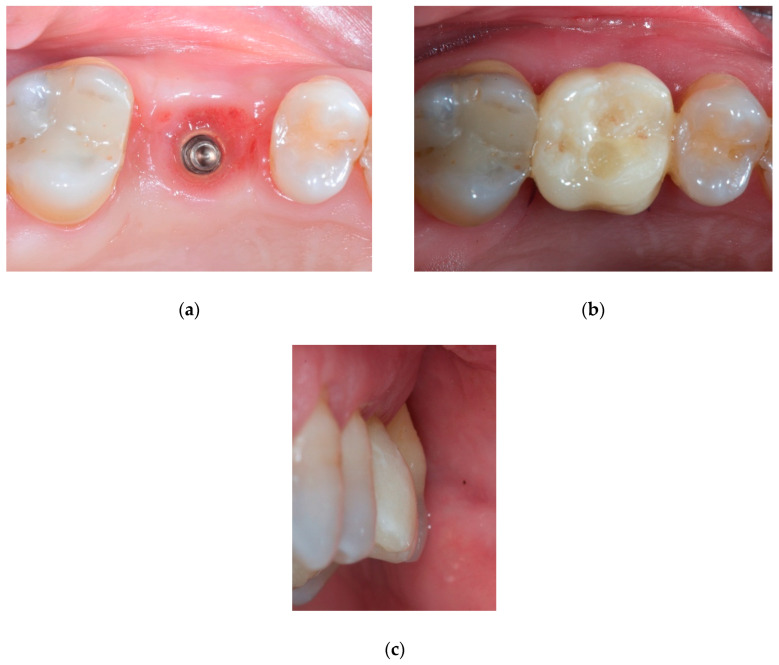
Intraoral view of the conditioned soft tissues after a healing period of 3 months: (**a**) occlusal view of the augmented peri-implant soft tissues shaped according to the emergence profile of the temporary crown. (**b**) Occlusal view of the augmented volume with the temporary crown in situ. (**c**) Buccal view of the augmented volume with the temporary crown in situ.

Soft tissues were conditioned for a period of 3 months (Figure 5a–c). Once the ideal architecture of the soft tissues was achieved, the definitive restoration was delivered to the patient.

## 3. Discussion

Soft tissue grafting is commonly indicated in case of insufficient keratinized gingiva or deficient gingival volume causing aesthetic imperfections and biological issues. The current literature considers autogenous soft tissue graft as the gold standard material, with the palate as the most frequent intraoral donor site, regardless of the surgical procedure. Zucchelli et al. compared with a randomized controlled design, sub-epithelial CTG versus de-epithelialized CTG and concluded that there were no differences in terms of discomfort, post-operative pain assessed by intake of analgesics, and bleeding [13]. The only statistically significant difference in the clinical outcomes between the two treatment groups was the greater increase in gingival thickness in those patients treated with de-epithelialized CTGs. The reason lies in the fact that sub-epithelial CTGs are harvested at a deeper level and contain less lamina propria and more fatty and glandular tissue. The authors also demonstrated that healing by secondary intention was not associated with increased post-operative discomfort. On the other hand, the corono-apical dimension of the graft and the residual soft-tissue thickness over the bone had a significant impact on the experienced post-operative pain. Nevertheless, the presence of a donor site necessarily leads to an increased rate of complications. A recent split-mouth cadaver study compared the risk of intra- and post-operative bleeding in relation to different palatal harvesting techniques [14]. The results highlighted how the trapdoor technique may significantly increase bleeding complications, due to a higher risk of damaging larger vessels during the harvesting procedure, which in turn leads to increased post-operative patient discomfort. According to Griffin et al., post-operative bleeding is more associated to post-surgical trauma and irritation of the surgical site rather than the operative procedure itself [15]. 

Thus, in order to reduce patients’ morbidity and discomfort, research activities have been directed toward the implementation of soft tissue substitutes. In this direction, the use of CMs showed promising results in terms of reduced post-operative pain and lower consumption of analgesics compared to autogenous CTGs [16]. Primarily, non-autogenous grafts must be biocompatible, allow and possibly promote the ingrowth of blood vessels and progenitor cells, maintain the original volume under mechanical forces generated by suturing, wound contraction, and mastication, prevent soft tissue shrinkage during the healing phase, and eliminate the need for a second surgical site to harvest autogenous tissue. In fact, wounds are subjected to contraction, infection and scar tissue formation. Autogenous grafts meet these requirements, however, disadvantages such as an additional surgical site, color mismatch and consistency, as well as a limited amount available for grafting, make these grafts less than ideal but still considered the gold standard [17]. Although many advantages have been reported in the use of xenogeneic CMs, only few data are available concerning the clinical indications and results. In a recent randomized controlled clinical trial, the comparison between the use of a three-dimensional CM and CTG yielded comparable results in terms of volumetric increase [16]. For both surgical procedures, post-operative complications were minimal. Histological analysis revealed well integrated grafts without foreign body reaction. Moreover, the volume-stable CM proved to be an excellent scaffold for the formation of new connective tissue. In another recent preclinical study, several parameters were evaluated regarding the host response to the insertion of a volume-stable CM [18]. The authors assessed, both in quantitative and qualitative terms, the inflammatory response and invasion of blood vessels and proliferating cells into the CM. The study demonstrated that at 90 days, the CM was fully integrated into newly formed soft connective tissue and the blood vessels were present in the biomaterial, emphasizing the biocompatibility of the CM. In addition, the results confirmed its comparability to CTGs in the treatment of soft tissue deficiencies. The question whether these comparable results can be maintained over the years has been answered by Thoma and colleagues in 2020 [19]. The authors, in the same population of patients treated in 2016, did not find any statistically significant differences in terms of peri-implant mucosal thickness and thickness changes between CM and autogenous subepithelial CTG at none of the study periods, namely six months, one year, and three years. Conversely, data over three years indicated an overall slight loss of the buccal contour between 0.2 mm and 0.13 mm for the CM and the CTG group, respectively; however, it was not possible to conclude whether such changes of the buccal contour were attributable to a loss of soft tissue, a loss of hard tissue or a combined loss of hard and soft tissue. In this matter, it is worth noting that CTGs, once transplanted, are encapsulated, which might lead to a delayed resorption and turnover, while the CM, in contrast, does not demonstrate any encapsulation, making it more prone to undergo remodeling processes [20]. This may explain why a higher shrinkage rate of CMs compared to CTGs may be observed over time. In the present report a CM has been used to re-establish a physiological buccal volume and to improve the implant site, minimizing at the same time the post-operative patient’s morbidity. This is in accordance with the indications currently suggested in literature [21], as the case showed herein had correct amounts of buccal bone and keratinized gingiva, but presented with an insufficient buccal soft tissue volume. The first surgical phase involved a split-thickness flap at the papillary level. The rationale is related to the fact that the flap slides buccally due to the volume increase following the placement of the CM. The split-thickness incision allows leaving a layer of intact periosteum intended to protect the exposed interproximal crestal bone underneath the mobilized papillae. The surgical papillae can then be sutured and adapted to the new prosthetic shape offered by the interim prosthesis. Once the buccal marginal bone is reached, the flap design is modified into a full-thickness incision in order to prepare the recipient space for the CM, avoiding releasing incisions. This simplified the surgical procedure and reduced intra- and post-operative complications, without compromising at the same time the final result. According to Thoma and coworkers [16], in order to reduce the invasiveness of the surgical procedure, the possibility of avoiding a second surgical site and the absence of releasing incisions greatly reduced the postoperative patient morbidity.

The results obtained in the present study strengthened the fact that volume-stable CMs may be used in place of autogenous soft tissue grafts to augment the missing volume at implant sites in the short term. This corroborates the findings of a previous study aimed to evaluate whether or not the use of volume-stable CMs may result in short-term soft tissue volume increase at implant sites non-inferior to autogenous subepithelial CTGs [16]. Thus, similarly to the present report, the authors included single-tooth implant sites in need of soft tissue volume increase. According to a randomized design, the sites were randomly assigned to receive either a cross-linked volume-stable CM or an autogenous subepithelial CTG. The authors concluded that the two treatments for soft tissue augmentation at implant sites rendered a similar gain in soft tissue volume, showing only minimal changes in soft tissue thickness between the first and last follow-up at 90 days. This complies favorably with the results obtained in the present report, where a satisfactory volumetric increase has been observed at the buccal aspect of a single implant at three months. Interestingly, although similar outcomes have been found between the present report and the study mentioned above, different surgical techniques have been adopted. Indeed, in the study by Thoma and colleagues, at the border between the ridge crest and the buccal aspect, a split thickness flap was prepared. Thereby, differently from the present report, the periosteum was not elevated on the buccal aspect to prepare a pouch. Clinically, dissecting and mobilizing a split-thickness flap is a more technique-sensitive and time-consuming procedure than creating a full-thickness envelope. In addition to an increased risk of flap perforation, partial-thickness flap dissection could excessively thin the overlying flap tissue and cause sloughing, necrosis, and compromised clinical results. These advantages have been already exploited in recession coverage procedures by means of volume-stable porcine CMs grafted into subperiosteal tunnels with successful results [22]. Therefore, according to the results obtained in this report, it is safe to assume that an adequate volumetric increase may be expected also in the case of a full-thickness buccal envelope which may be easier to prepare compared to a split-thickness pouch. This emphasizes the fact that volume-stable CMs can be effectively and safely used for soft tissue augmentation at implant sites regardless of the flap design used to prepare the recipient site. The critical point is the capability of the CM to maintain its volume after being grafted, particularly when positioned in a wet environment. Indeed, one could envisage that biomaterials used for soft tissue augmentation may collapse when placed in contact with liquids like blood. If a CM collapses after being grafted, host cell migration and blood vessel penetration may be hampered, which in turn negatively influences tissue integration and volume preservation. In this respect, in the present study, once implanted in the recipient site, the CM absorbed blood immediately but did not collapse, being able to preserve its original volume. As discussed in a recent animal study, this might be explained by the trabecular structure of the CM containing large and interconnected honeycomb-like pores that prevents the device to collapse while promoting the invasion of host cells in the biomaterial [11]. Another interesting aspect related to the present modified surgical procedure is related to the need of suturing reduced to a minimum. Only two mesial and distal single stitches were used to reposition the mesial and distal papilla apically according to the augmented buccal volume, with the temporary crown sustaining the volumetric increase of the soft tissues. This significantly reduced the pressure on the CM in the crestal region, which is normally caused by the primary wound closure, the location of the sutures, and excessively compressive prostheses. In this regard, it is worth noting that, in contrast to subepithelial CTGs, which are relatively resistant to mechanical load, CMs feature a high elasticity and appears to be more prone to compression forces. As a consequence of the modified flap design described herein, the CM was left to heal without any compression, leading to a successful volumetric increase at the buccal aspect. These findings correlate with those reported by Zeltner and coworkers, who noted that the volumetric increase was more pronounced in the buccal than in the crestal aspect, highlighting the importance to avoid any unnecessary pressure on the CM to achieve the desired soft tissue volume [21].

Undoubtedly, among the factors that determine the aesthetic quality of an implant-supported restoration, besides the quantity and quality of the soft tissues, the management of the proper prosthetic contour is known to play a crucial role [23]. According to this concept, the CM was positioned simultaneously with the temporary crown sustaining the graft, given that a correct emergence profile is essential to achieve a satisfying aesthetic result [24]. Indeed, the shape of the prosthetic emergence profile was able to condition the soft tissue maturation during the healing phase without excessive pressure. This explains why the volumetric increment of the soft tissues was complemented by the insertion of the temporary crown. It is authors’ opinion that without a customized emergence profile aimed to sustain and contour the maturation of the graft in a pressure-free environment, it would be more challenging to maintain the same volumes over time.

## 4. Conclusions

Within the limitations of a single case, the present report suggests that the use of a volume-stable porcine CM associated with a proper flap design can be considered as an alternative to autogenous CTG for buccal soft tissue augmentation. In addition to the advantages related to the absence of a donor site, this technique allowed obtaining satisfying functional and aesthetic results associated with marginal bone and soft tissue stability.

## Data Availability

No new data were created or analyzed in this study. Data sharing is not applicable to this article. The corresponding author remains available for any further clarification.
